# Surface Roughness and Bacterial Adhesion of CAD/CAM and Conventional Provisional Restorative Materials

**DOI:** 10.3390/ma19163421

**Published:** 2026-08-12

**Authors:** Hatice Defne Burduroglu, Burcu Kanpalta, Hilal Senturk, Zeynep Hale Keles, Soner Sismanoglu

**Affiliations:** 1 Department of Prosthodontics, Faculty of Dentistry, Istanbul University-Cerrahpaşa, 34098 Istanbul, Türkiye; defne.burduroglu@iuc.edu.tr; 2 Department of Prosthodontics, Faculty of Dentistry, Medipol University, 34214 Istanbul, Türkiye; burcu.kanpalta@medipol.edu.tr; 3 Department of Biomedical Engineering, Faculty of Engineering and Natural Sciences, Biruni University, 34020 Istanbul, Türkiye; hsenturk@biruni.edu.tr; 4 Department of Restorative Dentistry, Faculty of Dentistry, Istanbul Atlas University, 34408 Istanbul, Türkiye; 5 Department of Restorative Dentistry, Faculty of Dentistry, Istanbul University-Cerrahpaşa, 34098 Istanbul, Türkiye; soner.s@hotmail.com

**Keywords:** provisional restorative materials, surface roughness, bacterial adhesion, *Streptococcus mutans*, confocal laser microscopy

## Abstract

This study aimed to evaluate and compare the surface roughness (SR) and *Streptococcus mutans* adhesion of various provisional restorative materials fabricated using different manufacturing techniques, including traditional and computer-aided design/computer-aided manufacturing (CAD/CAM) methods. Disc-shaped specimens (n = 5) were prepared from four different materials: auto-polymerizing PEMA/PMMA (Dentalon Plus), bis-acrylic resin (Protemp 4) and two CAD/CAM PMMA blocks (Telio CAD, Vita CAD-Temp). All specimens were polished using a standardized protocol. SR was measured with a profilometer. For bacterial adhesion analysis, specimens were coated with artificial saliva to form a pellicle and incubated with *S. mutans* for 24 h. Adherent bacteria were quantified by confocal laser scanning microscopy and ImageJ/Fiji (version 2217 Stable) analysis. Group differences were analyzed using Welch’s ANOVA with Games–Howell post-hoc tests, and the SR–adhesion relationship was assessed by Spearman’s and Kendall’s correlation (α = 0.05). A strong, positive correlation was found between SR and bacterial adhesion (Spearman’s ρ = 0.803; Kendall’s τ = 0.656; *p* < 0.001). Dentalon Plus exhibited the highest SR and significantly greater bacterial adhesion than both CAD/CAM blocks. Although Telio CAD and Vita CAD-Temp had similarly low SR, Telio CAD showed significantly lower bacterial adhesion (*p* < 0.001), with a very large effect size. Thus, comparable surface roughness did not correspond to comparable bacterial adhesion, indicating that SR alone did not account for the observed differences. Within the limitations of this study, it was concluded that although SR is an important determinant of bacterial adhesion, it did not fully explain the differences observed between materials. Highly cross-linked PMMA CAD/CAM materials (Telio CAD) demonstrated superior resistance to *S. mutans* adhesion.

## 1. Introduction

The provisional fixed dental prosthesis fulfills several functions until the definitive cementation of the permanent restoration. Besides its fundamental functions of protecting pulpal vitality through thermal isolation and maintaining masticatory function, these provisional restorations offer valuable diagnostic insights [1]. Their ability to provide thermal isolation is crucial, protecting the prepared tooth from temperature changes that could otherwise increase sensitivity or lead to irreversible pulpal damage. Simultaneously, they ensure the patient can maintain chewing, which is vital for nutritional intake and overall well-being. These restorations also play a crucial role in enabling the careful assessment and accurate modification of the occlusal vertical dimension, the correction of irregular occlusal planes and optimizing force distribution. Crucially, provisional restorations serve as a vital aesthetic blueprint, enabling a collaborative assessment of prospective esthetic outcomes with the patient, allowing for necessary adjustments in shape, size, and shade prior to the final laboratory fabrication [2,3].

Moreover, clinical scenarios might necessitate the extended use of provisional restorations for prolonged durations. This requirement is particularly important in contexts such as the immediate loading of dental implants, where the provisional restoration provides an immediate aesthetic and functional solution during the osseointegration period, while simultaneously guiding the healing of soft tissues. Similarly, they are indispensable for the ongoing assessment of a tooth’s long-term prognosis, especially following complex endodontic treatment or in cases with compromised periodontal support, allowing the clinician to observe the tooth’s response over time before committing to a definitive restoration. They also play a pivotal role in the evaluation of a complex treatment plan’s efficacy, acting as a ‘trial restoration’ in reconstructive dentistry to confirm patient adaptation, phonetics, and overall comfort with proposed changes. In such applications, the provisional restoration’s material and structural integrity become paramount. It must exhibit exceptional durability and resilience to withstand sustained occlusal forces, resist wear, and maintain marginal integrity over months, or even years. Concurrently, its design must actively promote and sustain optimal periodontal health, featuring smooth, well-contoured surfaces to facilitate effective plaque control and prevent gingival inflammation, thereby ensuring the long-term success of the overall restorative process.

The long-term success of provisional restorations relies on acceptable marginal adaptation and surface properties that actively minimize bacterial adhesion. These features are not merely desirable; they are fundamental prerequisites for maintaining oral health, as bacterial adhesion and subsequent colonization represent the primary etiological factors for both periodontal disease and secondary caries. Salivary pellicle coats the natural surfaces such as enamel, dentine, oral mucosa, and also artificial surfaces of dental restorative materials [4]. It consists of salivary proteins, carbohydrates and lipids [5]. Following this pellicle coating, oral biofilm formation occurs beginning with early adhering bacteria like *Streptococcus mutans* (*S. mutans*), which are known as a major etiological agent of dental caries [6,7]. Further colonization of bacteria results in gingival inflammation and secondary caries adjacent to restorative materials [8,9]. This meticulous attention to the properties of provisional restorative materials thus not only supports the immediate stability of the restoration but also plays a pivotal role in preserving the long-term integrity of the tooth and the health of the surrounding periodontium during the entire course of treatment.

The surface roughness of dental restorative materials is a critical determinant influencing the formation and maturation of oral biofilm. Consequently, careful finishing and polishing procedures significantly impact the extent of bacterial adhesion and proliferation [10]. Polished surfaces impede bacterial adhesion and proliferation, rendering them susceptible to removal by brushing or saliva. Research has consistently demonstrated that a mean surface roughness exceeding 0.2 µm (Ra; Roughness Average) markedly promotes bacterial adherence and accelerates early plaque development [11,12]. Conversely, smooth surfaces render bacterial populations more susceptible to dislodgement through mastication, salivary flow, and, crucially, effective mechanical debridement via tooth brushing.

In contemporary restorative dentistry, many provisional restorative materials are available, each possessing distinct compositional and manufacturing characteristics that inherently influence their surface properties. These materials range from traditional auto-polymerizing polymethyl methacrylate (PMMA) and polyethyl methacrylate (PEMA) to more advanced options such as precisely machine-milled PMMA blocks, innovative resins designed for additive manufacturing (3D printers), light-curing urethane dimethacrylate (UDMA), and bis-acryl resins. The specific contents, including the monomer composition, type and percentage of filler particles, presence of cross-linking agents, and polymerization initiators, as well as the manufacturing techniques employed for each material, directly influence their inherent hardness, surface porosity, degree of monomer conversion, and susceptibility to degradation. These factors determine their eventual surface properties and, consequently, their biological interaction with the oral environment.

The clinical outcomes of bacterial accumulation on dental restoration surfaces are important, leading directly to the formation of dental plaque and, subsequently, gingival inflammation and secondary caries [13]. While extensive research has investigated the mechanisms of bacterial adhesion on definitive restorative materials such as amalgams, glass ionomers, composite resins, and ceramics [14,15,16], a noticeable scarcity exists in the literature investigating bacterial adherence on provisional restorative materials. Particularly for recently introduced and rapidly adopted fabrication technologies, including 3D-printed resins and computer-aided design/computer-aided manufacturing (CAD/CAM) resin blocks [3,13]. Given that provisional restorations are fundamentally designed to preserve the health and integrity of the oral tissues throughout the treatment phase, minimizing bacterial adherence on their surfaces is of paramount importance. Despite this clinical importance, the available evidence on provisional restorative materials is not only limited but also difficult to compare, as most studies address a single material class and rely on surface roughness as the principal explanatory variable. Consequently, it remains unclear whether surface roughness alone accounts for the differences in bacterial adhesion observed among contemporary provisional restorative materials, or whether materials of comparable roughness may nevertheless differ in this respect. This distinction is clinically meaningful, because roughness can be modified chairside through finishing and polishing, whereas composition and the degree of industrial polymerization are inherent to the material and cannot be altered by the clinician. Directly comparing conventional, chairside-processed materials with industrially polymerized CAD/CAM blocks under identical conditions therefore offers an opportunity to examine whether surface roughness alone accounts for the differences observed, which has rarely been done for provisional restorative materials. Therefore, this study aims to investigate and compare the adhesion properties of *S. mutans*, a key cariogenic bacterium, on various provisional restorative materials fabricated using different techniques. The first null hypothesis was that all provisional restorative materials tested would exhibit similar bacterial adhesion. The second null hypothesis was that surface roughness would have no effect on bacterial adhesion.

## 2. Materials and Methods

### 2.1. Specimen Preparation

The provisional restorative materials, their composition and manufacturers used in the study are listed in Table 1.

Disc-shaped specimens were prepared with dimensions of 10 mm diameter and 2 mm thickness (n = 5). A precision saw was used to prepare the CAD/CAM block specimens, and a diamond burr coupled to a rotating handpiece was used to cut the blocks into a disc shape. Two glass plates and a specially made silicon mold were used to fabricate specimens which are produced by mixing components. Materials were mixed according to the manufacturer’s instructions and packed into the custom silicon mold. The silicon mold with packed material was then compressed with two glass plates from both sides and allowed for polymerization. All polymerized specimens were polished with 400, 800, 1200 and 2400 grit SiC sandpaper respectively by a single operator. After polishing, the specimens were stored in 25 °C distilled water for 1 week.

### 2.2. Surface Roughness Measurement

A profilometer (Perthometer M2; Mahr GmbH, Göttingen, Germany) was used to measure surface roughness (Ra), with a cut-off length of 0.25 mm and a tracing length of 1.75 mm. Three consecutive readings were made by turning the position of the profilometer 120° clockwise each time and an average value was calculated.

The specimens were ultrasonically cleaned and sterilized in an autoclave at 121 °C for 15 min. Then, the specimens were washed with 5 mL of saline and placed into sterilized Petri dishes.

### 2.3. Bacterial Culture and Adhesion Procedure

The *S. mutans* bacteria obtained from stock (UA159; ATCC 700610, American Type Culture Collection, Manassas, VA, USA) were plated on Columbia agar and incubated for 24 h at 37 °C in a 10% CO_2_ atmosphere environment. The cultured bacteria were then transferred into falcon tubes with 5 mL Brain Heart Infusion (BHI) and incubated at 37 °C in a 10% CO_2_ environment for 18 h. The falcon tubes were centrifuged for 5 min at a speed of 4000 rpm and the supernatant was discarded. The pellet was resuspended with 1× PBS and mixed by pipetting up and down. Tubes were centrifuged again for 5 min. Pellets were resuspended with 300 µL 1× PBS, then transferred into 96-well plates. Bacterial density was measured at 600 nm and diluted with PBS to obtain a final concentration of 1 × 10^9^ CFU/mL.

The acquired pellicle was formed using a saliva substitute (Biotene; GSK, Brentford, UK) supplemented with porcine gastric mucin (Type II; Sigma-Aldrich, St. Louis, MO, USA) at a concentration of 850 mg/L, corresponding to the salivary mucin concentration reported by Hahnel et al. [17]. Five milliliters of the suspension was added to each specimen placed in a sterile Petri dish, and the specimens were kept for 1 h to allow pellicle formation. The specimens were then rinsed with 5 mL of sterile saline and transferred into sterile Falcon tubes.

Five ml bacterial suspension which was prepared previously was added to each specimen in falcon tubes and left for 15 min. A sterilized mixture of BHI and 5% sucrose was then added to cover all specimens. The specimens with bacterial suspension were incubated at 37 °C in a 5% CO_2_ environment for 24 h. After that, the samples were washed with 1× PBS and mounted on slides for confocal microscopic analysis.

### 2.4. Confocal Laser Scanning Microscopy and Quantification

Fluorescein diacetate (FDA; Sigma-Aldrich, St. Louis, MO, USA) and ethidium bromide (EtBr; 0.07% solution, Sigma-Aldrich, St. Louis, MO, USA) were used to analyze bacterial adherence on specimens. FDA and EtBr were mixed with 1 mL of cold saline. This mixture was added to each specimen and covered with a glass coverslip.

**Figure 1:**
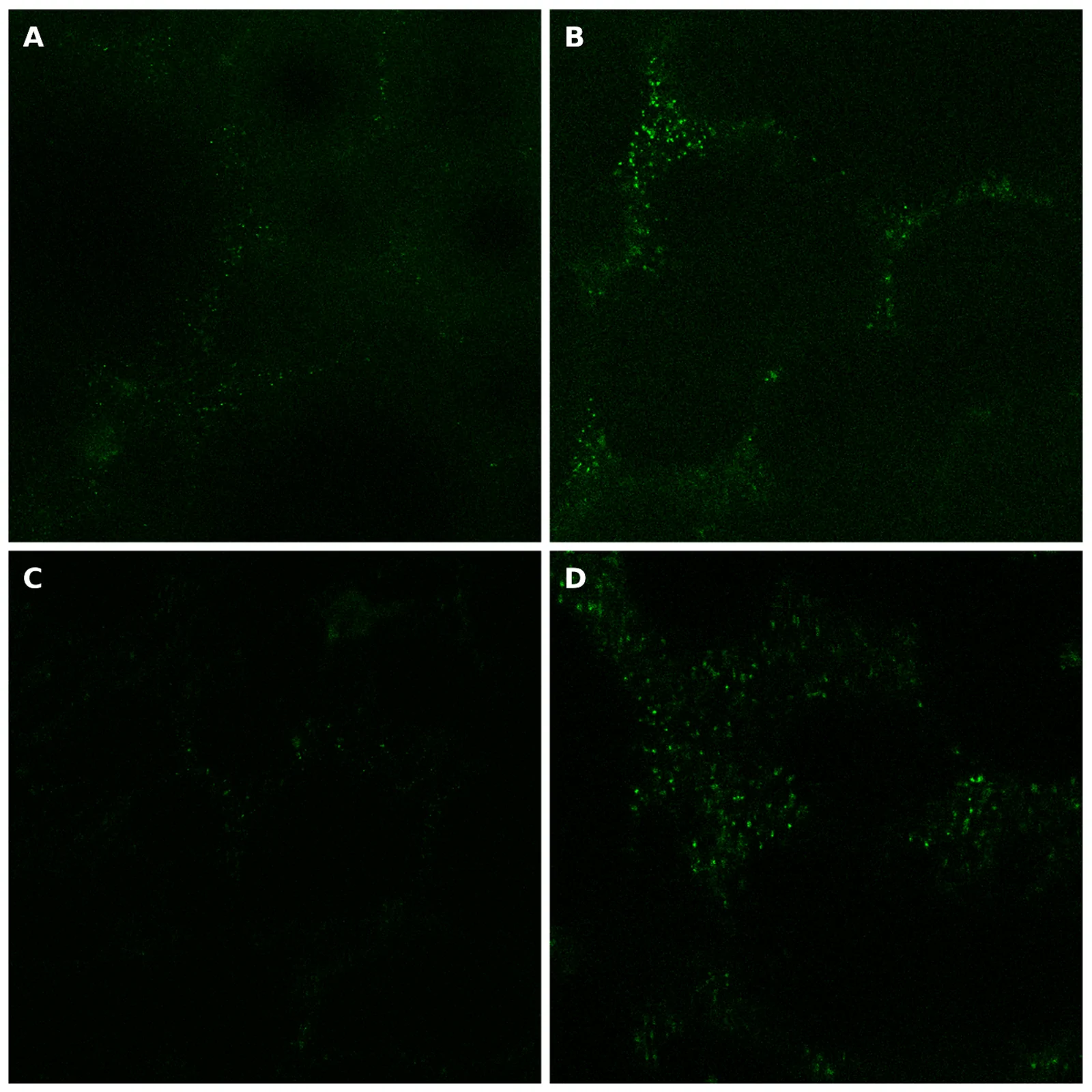
Representative confocal laser scanning microscopy images of *Streptococcus mutans* adhesion on the tested provisional restorative materials after 24 h of incubation. (**A**) Dentalon Plus, (**B**) Protemp 4, (**C**) Telio CAD, (**D**) Vita CAD-Temp. Adherent bacteria appear as green fluorescence. Dense bacterial colonization is evident on the conventional materials (**A**,**B**) and Vita CAD-Temp (**D**), whereas Telio CAD (**C**) shows markedly sparser adhesion, consistent with the quantitative findings.

A confocal laser microscope (Leica TCS SP8; Wetzlar, Germany) was used to visualize and quantify adherent bacteria on the specimens. Stained bacteria images were collected from the specimens using a 63× oil-immersion objective, with FDA and EtBr fluorescence recorded in separate channels using the excitation and emission settings recommended by the manufacturer for each fluorophore. The confocal scans were saved as images and used to quantify adherent bacteria using Image-J/Fiji (version 2217 Stable; National Institutes of Health, Bethesda, MD, USA). Representative confocal images of each material are shown in Figure 1.

### 2.5. Statistical Analysis

All statistical analyses were performed using IBM SPSS Statistics (version 31.0; IBM Corp., Armonk, NY, USA), with the significance level set at α = 0.05. Descriptive statistics (mean ± standard deviation) were calculated for surface roughness (SR) and bacterial adhesion (BA) of each material group (n = 5 per group).

A sensitivity power analysis (G*Power 3.1.9.7; F tests, one-way ANOVA, fixed effects; four groups, total N = 20, α = 0.05) indicated that the design provided 80% power to detect large effects (Cohen’s f ≥ 0.84; η^2^ ≥ 0.41). The effect sizes observed for both surface roughness (f = 1.22) and bacterial adhesion (f = 7.77) exceeded this threshold, indicating that the sample was adequate to detect the between-material differences reported here.

Prior to comparative analysis, the assumptions of normality and homogeneity of variances were assessed using the Shapiro–Wilk and Levene’s tests, respectively. Given the small sample size and to provide a conservative and consistent approach across both variables, group differences were evaluated using Welch’s analysis of variance (ANOVA), which does not assume equal variances, followed by the Games–Howell post-hoc test for pairwise comparisons. Pairwise effect sizes were expressed as Cohen’s d.

The relationship between SR and BA was examined across all individual specimens (N = 20) using Spearman’s rank-order correlation coefficient (ρ). Because SR values were distributed across a limited number of discrete levels, Kendall’s τ was additionally computed to account for tied ranks. To determine whether the association was driven by differences between materials rather than by variation within them, Spearman’s correlation was additionally computed separately within each material group. Both coefficients are reported with their corresponding two-tailed significance values.

## 3. Results

**Figure 2:**
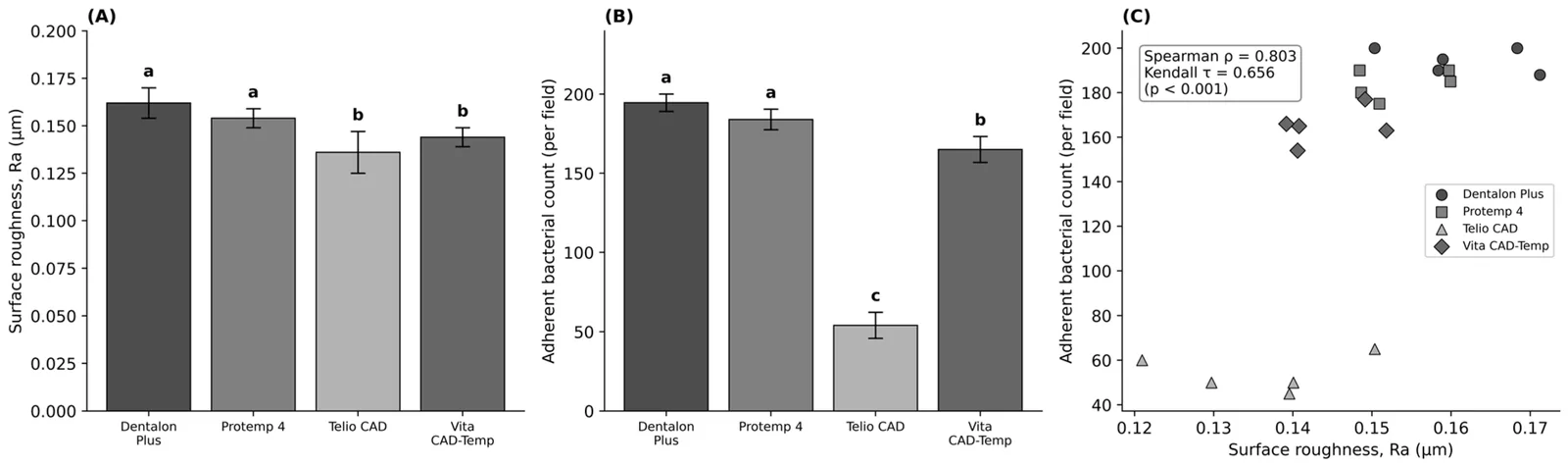
Surface roughness and bacterial adhesion of the four tested provisional restorative materials. (**A**) Mean surface roughness (Ra, µm) and (**B**) mean adherent bacterial count per field (n = 5 per group); error bars represent standard deviations, and bars sharing the same lowercase letter are not significantly different (Games–Howell post-hoc test following Welch’s ANOVA, *p* > 0.05). Bars not sharing a common letter differ significantly (*p* < 0.05), and letters were assigned separately within each panel. (**C**) Relationship between surface roughness and adherent bacterial count across all individual specimens (N = 20), with materials distinguished by color and symbol; the correlation was strong, positive and statistically significant (Spearman’s ρ = 0.803; Kendall’s τ = 0.656; *p* < 0.001). Telio CAD and Vita CAD-Temp showed comparable surface roughness but markedly different bacterial adhesion, illustrating the contribution of material composition beyond surface roughness alone.

Descriptive statistics and pairwise comparisons for surface roughness (SR) and bacterial adhesion (BA) are presented in Table 2 and Table 3, respectively, and illustrated in Figure 2.

### 3.1. Surface Roughness

Welch’s ANOVA revealed a significant effect of material on SR (F(3, 8.6) = 7.65, *p* = 0.008) (Table 2; Figure 2A). Dentalon Plus exhibited the highest mean SR (0.162 ± 0.008 µm), followed by Protemp 4 (0.154 ± 0.005 µm), Vita CAD-Temp (0.144 ± 0.005 µm) and Telio CAD (0.136 ± 0.011 µm). Games–Howell post-hoc comparisons showed that Dentalon Plus had significantly higher SR than both Telio CAD (*p* = 0.017, d = 2.60) and Vita CAD-Temp (*p* = 0.021, d = 2.55). No significant differences were found among the remaining pairs, including the two conventional materials (Dentalon Plus vs. Protemp 4, *p* = 0.353) and the two CAD/CAM materials (Telio CAD vs. Vita CAD-Temp, *p* = 0.537). Thus, the CAD/CAM materials formed one homogeneous low-roughness group and the conventional materials a higher-roughness group, with a partial overlap between Protemp 4 and the CAD/CAM blocks.

### 3.2. Bacterial Adhesion

Welch’s ANOVA showed a highly significant effect of material on BA (F(3, 8.8) = 316.60, *p* < 0.001) (Table 3; Figure 2B). The highest adhesion was observed for Dentalon Plus (194.6 ± 5.5 bacteria/field) and Protemp 4 (184.0 ± 6.5), which did not differ significantly from each other (*p* = 0.094). Vita CAD-Temp showed an intermediate value (165.0 ± 8.2), significantly lower than both conventional materials (vs. Dentalon Plus, *p* = 0.001; vs. Protemp 4, *p* = 0.017). Telio CAD exhibited by far the lowest bacterial adhesion (54.0 ± 8.2) and differed significantly from all other materials, with very large effect sizes (vs. Dentalon Plus, *p* < 0.001, d = 20.06; vs. Protemp 4, *p* < 0.001, d = 17.53; vs. Vita CAD-Temp, *p* < 0.001, d = 13.51). Consequently, the four materials separated into three distinct adhesion levels: high (Dentalon Plus, Protemp 4), intermediate (Vita CAD-Temp) and low (Telio CAD). These differences were also visually apparent in the representative confocal images (Figure 1).

### 3.3. Correlation Between Surface Roughness and Bacterial Adhesion

Spearman’s rank-order correlation, computed across all individual specimens (N = 20), revealed a strong, positive and statistically significant relationship between SR and BA (Spearman’s ρ = 0.803, *p* < 0.001) (Figure 2C). Because surface roughness values were distributed across a limited number of discrete levels, Kendall’s τ was additionally calculated and confirmed the association (Kendall’s τ = 0.656, *p* < 0.001), indicating that higher surface roughness was generally associated with greater bacterial adhesion. Notably, this overall trend did not fully account for the behavior of Telio CAD and Vita CAD-Temp, which showed statistically comparable surface roughness (*p* = 0.537) yet markedly different bacterial adhesion (*p* < 0.001), suggesting that factors beyond surface roughness contributed to bacterial adhesion. When computed separately within each material group, no significant correlation between SR and BA was observed (ρ ranging from −0.35 to 0.44; all *p* > 0.45), indicating that the pooled association primarily reflected differences between materials rather than a within-material relationship between roughness and adhesion.

## 4. Discussion

This study evaluated the surface roughness and *S. mutans* adhesion of four provisional restorative materials fabricated by conventional and CAD/CAM techniques. The tested materials differed significantly in both parameters: the conventional materials (Dentalon Plus, Protemp 4) exhibited higher surface roughness and greater bacterial adhesion, whereas the CAD/CAM PMMA blocks showed the lowest values. Although Telio CAD and Vita CAD-Temp presented comparable, low surface roughness, Telio CAD attracted markedly fewer bacteria, indicating that roughness alone did not account for the difference. A strong positive correlation was found between surface roughness and bacterial adhesion overall. Accordingly, the first null hypothesis, that all tested provisional restorative materials would exhibit similar bacterial adhesion, was partly rejected, and the second null hypothesis, that surface roughness would have no effect on bacterial adhesion, was rejected.

The relationship between the surface roughness of dental materials and bacterial adherence is well established [11,13,18]. Intraoral hard surfaces attract a variety of microorganisms [14,19], and surface roughness has a significant effect on bacterial adhesion [20]. Initial bacterial adhesion occurs preferentially on roughened surfaces, where bacteria are shielded from salivary flow and masticatory forces [21]. It is widely accepted that roughness values below approximately 0.2 µm do not further influence bacterial adhesion on dental hard surfaces [12,22]. This threshold effect has gained renewed relevance with the introduction of CAD/CAM and additively manufactured provisional restorative materials, for which comparative adhesion data remain limited but are rapidly accumulating [23,24]. In the present study, the quantitative assessment revealed a strong, positive and statistically significant correlation between surface roughness and the initial retention of oral bacteria, a key precursor of plaque accumulation. It should be noted that the pooled correlation reported here is driven by differences between materials: within each material group, where the range of surface roughness was narrow, no association between roughness and bacterial adhesion was detectable. The correlation therefore describes a general trend across materials of differing roughness rather than a within-material dependence of adhesion on roughness.

The role of surface roughness in bacterial colonization is well established but complex. Several authors have reported that once surfaces fall below the ~0.2 µm threshold, factors other than roughness begin to govern bacterial adhesion [12,22]. While a positive correlation between roughness and early bacterial adhesion persists at higher roughness values, this relationship weakens as surfaces become progressively smoother [25]. Under these conditions, polymer-based materials with nearly identical, low Ra values can still exhibit markedly different bacterial adhesion, indicating that material-specific properties become dominant at this scale [12,26]. Consistent with this, in the present study Telio CAD and Vita CAD-Temp displayed statistically comparable surface roughness yet significantly different bacterial adhesion, indicating that factors other than surface topography contributed to this difference.

It is noteworthy that all four materials exhibited mean surface roughness values below the ~0.2 µm threshold generally considered clinically relevant for plaque retention [11,22], yet they still differed significantly in both roughness and bacterial adhesion. This indicates that, even among materials that would all be regarded as clinically “smooth”, meaningful differences in early bacterial colonization can persist. In this study, Dentalon Plus exhibited the highest surface roughness and, correspondingly, the greatest bacterial adhesion, differing significantly from both CAD/CAM blocks. This is in line with reports that surface roughness within the 0.06–0.2 µm range positively affects early bacterial adhesion [12]. However, our results also showed that materials with comparable roughness can exhibit significantly different adhesion: Telio CAD attracted far fewer bacteria than Vita CAD-Temp (*p* < 0.001), despite statistically similar, low surface roughness (*p* = 0.537). This dissociation between roughness and adhesion indicates that bacterial retention is not governed by topography alone; material composition and surface physicochemistry have been proposed as contributing factors [12,26]. Notably, the superior performance of milled PMMA is not limited to in vitro conditions; a recent split-mouth clinical trial reported significantly lower *S. mutans* accumulation on milled PMMA provisional restorations than on conventional acrylic resin, supporting the clinical relevance of our findings [27]. Conversely, the two conventional materials, despite their different chemistries (auto-polymerizing PEMA/PMMA in Dentalon Plus and bis-acrylic resin in Protemp 4), showed comparably high surface roughness and did not differ significantly in bacterial adhesion. This suggests that in the higher-roughness range, surface topography remained the predominant factor governing adhesion, overriding compositional differences that become influential only once surfaces are sufficiently smooth. From a clinical perspective, these findings are particularly relevant for provisional restorations intended for extended service, such as during implant osseointegration or prolonged treatment planning. In such situations, where the restoration must resist bacterial colonization over months rather than days, selecting a material with inherently low adhesion, such as highly cross-linked milled PMMA, may offer a more durable biological advantage than surface polishing alone, since polishing effects can diminish over time through wear and degradation.

Beyond roughness, dental plaque formation is influenced by several physicochemical characteristics of the restorative material, including surface free energy, hydrophobicity/wettability and surface electrochemistry. Brentel et al. emphasized that finishing and polishing procedures can alter both surface free energy and roughness, thereby promoting or hindering biofilm development [10]. According to the thermodynamic approach to adhesion, bacterial strains with high surface free energy, such as *S. mutans*, adhere preferentially to substrates that also possess high surface free energy [28]. The relative contribution of these parameters, however, remains debated: while Yuan et al. reported that surface free energy influences early adhesion on smooth surfaces, they found hydrophobicity to be less relevant at this initial stage [12]. More recent evidence further illustrates this complexity, as a smoother and more wettable CAD/CAM material was shown to accumulate significantly fewer *S. mutans* than rougher, less wettable composites, indicating that wettability and microstructure can outweigh roughness in determining adhesion [26]. As surface free energy, contact angle and monomer release were not measured in the present study, the influence of these factors on our findings remains to be confirmed in future work.

Polymer-based dental materials typically consist of a hydrophobic resin matrix and hydrophilic filler particles, producing a chemically heterogeneous surface whose properties depend strongly on polymer composition and on the size and shape of the fillers [12,29]. Surface chemistry is particularly important when released components affect the adjacent microbial population. Hansel et al. reported that certain resin monomers, such as triethyleneglycol dimethacrylate or methacrylic acid, can modulate bacterial growth or even stimulate caries-associated species such as *S. sobrinus* and *L. acidophilus* [30]. Accordingly, bacterial accumulation has been shown to vary with both filler size and the specific matrix monomer employed [31].

In the present study, the superior resistance of Telio CAD to bacterial adhesion, despite roughness comparable to the other CAD/CAM block, may be related to its manufacturing process and structural homogeneity. Telio CAD consists of cross-linked PMMA with a filler-free composition (PMMA 99.5 wt%), whereas VITA CAD-Temp is a microfiller-reinforced polyacrylic in which inorganic microfillers are polymerized into the network. Both manufacturers describe their blocks as cross-linked and structurally homogeneous, so that the two materials differ primarily in the presence of an inorganic filler phase rather than in declared homogeneity. Even when such fillers produce a smooth surface, the filler particles and the filler–matrix interface have been proposed to act as microscopic niches for pellicle accumulation and subsequent bacterial attachment, an effect that would not arise on a filler-free surface [29]. This possibility could not be tested here, as the surface microstructure of the specimens was not examined. In addition, the high degree of cross-linking in Telio CAD is expected to yield a more stable surface with reduced monomer release, which may further limit bacterial stimulation [30]. This interpretation is consistent with the broader literature: milled CAD/CAM PMMA has repeatedly been reported to accumulate less microbial biomass than conventional and additively manufactured resins, both in vitro and in a systematic review [23,24]. Overall, while surface roughness remained decisive for rougher materials such as Dentalon Plus, the low bacterial adhesion of Telio CAD cannot be explained by topography alone. The mechanisms outlined above are offered as possible explanations consistent with the manufacturers’ compositional data and with the existing literature; as neither chemical composition, degree of conversion, nor structural homogeneity was assessed in the present study, they remain hypotheses that require direct experimental verification.

Some limitations of this study should be considered when interpreting the findings. As an in vitro model, it could not fully reproduce the dynamic conditions of the oral cavity, where salivary flow, dietary intake and a multispecies microbial community continuously modulate biofilm formation; adhesion was assessed for a single early colonizer, *S. mutans*, over a 24-h period. In addition, the surface microstructure of the specimens was not examined by scanning electron microscopy, atomic force microscopy or three-dimensional profilometry, and surface free energy, contact angle and monomer release were not quantified. The proposed compositional and microstructural mechanisms therefore remain interpretations rather than direct measurements. Surface roughness was moreover characterized only by Ra, an arithmetic mean parameter that does not capture surface morphology, porosity or filler exposure, and no additional amplitude or spatial roughness parameters were recorded. The relationship between surface roughness and bacterial adhesion was assessed correlationally, and the present design does not permit the observed differences to be attributed causally to any individual surface property. Finally, the relatively small sample size warrants some caution, although the very large effect sizes observed for bacterial adhesion suggest that the main differences are robust. Future studies incorporating multispecies biofilms, longer observation periods and direct characterization of surface physicochemistry would help clarify the mechanisms underlying the material-dependent differences reported here.

## 5. Conclusions

Within the limitations of this in vitro study, surface topography alone did not account for the bacterial adhesion observed on the provisional restorative materials tested. The following conclusions can be drawn:

1. A strong, positive correlation was found between surface roughness and *S. mutans* adhesion (Spearman’s ρ = 0.803), indicating that greater surface roughness was generally associated with greater bacterial adhesion. This association reflected differences between materials rather than a relationship detectable within individual material groups.
2. Surface roughness alone did not fully explain adhesion behavior. Despite comparable surface roughness, Telio CAD showed markedly lower adhesion than Vita CAD-Temp, indicating that on smooth surfaces determinants other than roughness govern bacterial adhesion.
3. Telio CAD, a milled CAD/CAM PMMA block, was the most resistant to early *S. mutans* adhesion, and its selection may help limit early biofilm formation.

## Figures and Tables

**Table 1:** The tested materials, compositions, batch numbers and manufacturers of the test groups.

Materials	Type	Composition	Batch Number
Dentalon Plus; Heraeus Kulzer, Hanau, Germany.	Auto-polymerizing PEMA/PMMA	Powder: polyethyl methacrylate/polymethyl acrylate. Liquid: n-butyl methacrylate, urethane acrylate, ethyl methacrylate	010215/010293
Protemp 4; 3M ESPE, Neuss, Germany.	Auto-polymerizing bis-acrylic composite	Ethoxylated bisphenol-A dimethacrylate (CAS 41637-38-1) 45–55 wt%; surface-modified amorphous silica (CAS 7631-86-9) 20–30 wt%; DESMA (CAS 1101874-33-2) 10–15 wt%; silane-treated silica (CAS 68909-20-6) 5–15 wt%	555927
Telio CAD; Ivoclar Vivadent, Schaan, Liechtenstein	Milled cross-linked PMMA block	PMMA 99.5 wt%; pigments < 1.0 wt%	T20206
CAD-Temp; Vita Zahnfabrik, Bad Sackingen, Germany	Milled microfiller-reinforced polyacrylic (MRP) block	Cross-linked, fiber-free, methyl methacrylate-free acrylate polymer with inorganic microfiller polymerized into the network	45870/34760

Compositional data as declared by the manufacturers in the respective safety data sheets and instructions for use. Abbreviations: PEMA, poly(ethyl methacrylate); PMMA, poly(methyl methacrylate); DESMA, reaction products of 1,6-diisocyanatohexane with 2-[(2-methacryloyl)ethyl]-6-hydroxyhexanoate and 2-hydroxyethyl methacrylate.

**Table 2:** Surface roughness (Ra, mean ± SD) of the tested provisional restorative materials: descriptive statistics, Games–Howell pairwise comparisons and effect sizes following Welch’s ANOVA (F(3, 8.6) = 7.65, *p* = 0.008).

Material	Ra (µm)	Comparison	*p*-Value	Cohen’s d
Dentalon Plus	0.162 ± 0.008 ^a^	Dentalon vs. Protemp 4	0.353	1.13
Protemp 4	0.154 ± 0.005 ^a^	Dentalon vs. Telio CAD	0.017 *	2.60
Telio CAD	0.136 ± 0.011 ^b^	Dentalon vs. Vita CAD-Temp	0.021 *	2.55
Vita CAD-Temp	0.144 ± 0.005 ^b^	Protemp 4 vs. Telio CAD	0.072	2.01
		Protemp 4 vs. Vita CAD-Temp	0.078	1.83
		Telio CAD vs. Vita CAD-Temp	0.537	0.89

Values sharing the same superscript letter are not significantly different (*p* > 0.05). * Significant at *p* < 0.05.

**Table 3:** Bacterial adhesion (adherent bacterial count per field, mean ± SD) of the tested provisional restorative materials: descriptive statistics, Games–Howell pairwise comparisons and effect sizes following Welch’s ANOVA (F(3, 8.8) = 316.60, *p* < 0.001).

Material	Count/Field	Comparison	*p*-Value	Cohen’s d
Dentalon Plus	194.6 ± 5.5 ^a^	Dentalon vs. Protemp 4	0.094	1.75
Protemp 4	184.0 ± 6.5 ^a^	Dentalon vs. Telio CAD	<0.001 *	20.06
Telio CAD	54.0 ± 8.2 ^c^	Dentalon vs. Vita CAD-Temp	0.001 *	4.22
Vita CAD-Temp	165.0 ± 8.2 ^b^	Protemp 4 vs. Telio CAD	<0.001 *	17.53
		Protemp 4 vs. Vita CAD-Temp	0.017 *	2.56
		Telio CAD vs. Vita CAD-Temp	<0.001 *	13.51

Values sharing the same superscript letter are not significantly different (*p* > 0.05). * Significant at *p* < 0.05.

## Data Availability

The original contributions presented in this study are included in the article. Further inquiries can be directed to the corresponding author.
